# Retraction: A mixed methods analysis of the magnitude and associated factors of time management practice among primary hospital employees in North Gondar, Ethiopia

**DOI:** 10.1371/journal.pgph.0005476

**Published:** 2025-11-18

**Authors:** The PLOS Global Public Health Editors

This article [1] was identified as one of a series of submissions for which we have concerns about authorship and adherence to the journal’s first and second publication criteria.

In addition, the article [1] reports the same study and results as a previously published article [2, Expression of Concern in 3] that was not cited or discussed in [1].

In light of these issues, the *PLOS Global Public Health* Editors retract this article.

All authors either did not respond directly or could not be reached.
